# Reassessing the Use of Membranes in Peri-Implantitis Surgery: A Systematic Review and Meta-Analysis of In Vivo Studies

**DOI:** 10.3390/jfb16070262

**Published:** 2025-07-15

**Authors:** Young Joon Cho, Yong Tak Jeong, Hyun Nyun Woo, Hyun Woo Cho, Min Gu Kang, Sung-Min Hwang, Jae-Mok Lee

**Affiliations:** 1 Mac Dental Clinic, Daegu 42117, Republic of Korea; macdental@knu.ac.kr (Y.J.C.); makai29@knu.ac.kr (Y.T.J.); 2 Department of Periodontology, School of Dentistry, Kyungpook National University, Daegu 41940, Republic of Korea; 3 Private Practice, New York, NY 10075, USA; hw2719@cumc.columbia.edu; 4 Department of Oral & Maxillofacial Surgery, Boston University Henry M. Goldman School of Dental Medicine, Boston, MA 02118, USA; dancho99@bu.edu; 5 Private Practice, Gumi-si 39236, Kyeongsangbuk-do, Republic of Korea; dent0000@hanmail.net

**Keywords:** peri-implantitis, detoxification, guided bone regeneration, resorbable membranes, non-resorbable membranes, bone regeneration, reosseointegration

## Abstract

Peri-implantitis (PI) presents a growing challenge in implant dentistry, with regenerative surgical approaches often incorporating barrier membranes despite the uncertainty of their clinical value. This systematic review and meta-analysis of in vivo studies aimed to evaluate the efficacy of barrier membranes in the reconstructive surgical treatment of PI. A comprehensive electronic search was performed in PubMed, Scopus, Google Scholar, and the Cochrane Library, covering studies published from 1990 to 2024. The protocol followed PRISMA guidelines and was registered in PROSPERO (CRD42025625417). Eligible studies included in vivo investigations comparing regenerative procedures with and without membrane use, with a minimum follow-up of 6 months and at least 10 implants per study. Risk of bias (RoB) was assessed using the Cochrane RoB tool. The meta-analysis was conducted using a random-effects model and included 15 studies comprising 560 patients. Although not consistently statistically significant, the findings suggested that membrane use may offer enhanced outcomes in terms of probing pocket depth (PPD) reduction and marginal bone level (MLB) gain. The evidence was limited by high clinical heterogeneity, variability in outcome definitions, and short follow-up durations. While membranes are commonly utilized, current evidence does not justify their routine use. Further well-designed, long-term clinical trials are needed to establish specific indications and optimize treatment strategies.

## 1. Introduction

Peri-implantitis (PI) is a serious pathological condition triggered by the accumulation of dental plaque around dental implants, leading to the progressive loss of supporting bone and inflammation of the peri-implant mucosa [1,2]. The primary culprits include the bacterial biofilm within dental plaque and its associated pathogenic byproducts, such as lipopolysaccharides and proteolytic enzymes, which trigger a destructive host immune response, particularly in individuals with poor plaque control and irregular dental attendance [3]. Anti-infective treatment approaches, such as mechanical debridement, implantoplasty, and the application of local antibiotics, can mitigate soft tissue inflammation and decelerate the disease’s progression [4]. However, once PI takes hold, achieving full detoxification of the infected implant surface is an arduous task, as is restoring the damaged bone to support implant reosseointegration [2,5].

Emerging techniques utilizing scaffolds show promise in regenerating periodontal and peri-implant tissues [6], yet there is still no universally effective and clearly defined treatment protocol. Similar to periodontitis treatment, PI management involves both non-surgical and surgical strategies [7]. Surgical treatment is further classified into resective procedures, which focus on eliminating inflamed tissues and reshaping bony defects, and reconstructive procedures, which aim to regenerate lost peri-implant structures using bone grafts and/or membranes, reflecting the ultimate therapeutic goal in PI management [5,8]. The representative regenerative technique, guided bone regeneration (GBR), has been utilized for an extended period [9,10]. Although membranes are used to prevent soft tissue interference and support bone regeneration, their clinical effectiveness is questioned due to inconsistent healing environments and lingering infections [2,7].

We employ a systematic review and meta-analysis approach to explore the latest advancements in procedures and membranes for PI therapy, assessing their efficacy through clinical trials. By synthesizing data from multiple studies, we aim to provide a comprehensive overview of the benefits and limitations of membranes used in PI treatment. This approach also offers insights into future perspectives and helps establish evidence-based guidelines for clinical practice.

## 2. Materials and Methods

### 2.1. Study Design

This systematic review evaluated the effect of barrier membranes in the regenerative surgical treatment of PI by comparing procedures involving bone grafts (BGs) with or without the adjunctive use of barrier membranes, assessing clinical outcomes such as probing pocket depth (PPD) reduction, marginal bone level (MLB) gain, and clinical attachment level (CAL) gain.

### 2.2. Protocol and Registration

**Figure 1:**
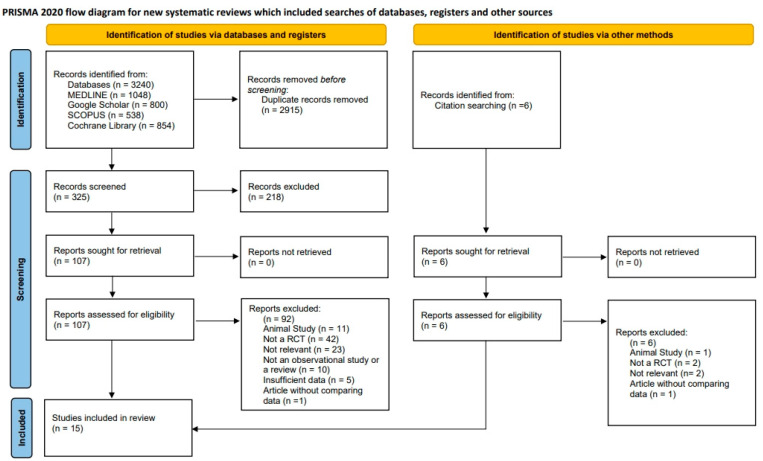
Preferred Reporting Items for Systematic Reviews and Meta-Analyses (PRISMA) flow diagram representing study selection and inclusion.

This review followed the Preferred Reporting Items for Systematic Reviews and Meta-Analyses (PRISMA) guidelines (Figure 1). As this study was based solely on previously published data, ethical approval and patient consent were not required. The review was registered with the International Prospective Register of Systematic Reviews (PROSPERO; registration ID: CRD42025625417). No formal protocol was prepared beyond the PROSPERO registration, and no amendments were made to the registered information. The registration entry includes the study objective, inclusion and exclusion criteria, and planned outcomes.

### 2.3. PICO

#### 2.3.1. Population

The population consisted of patients diagnosed with PI, characterized by clinical signs (e.g., bleeding on probing (BOP), suppuration, increased PPD) and radiographic evidence of MLB loss around osseointegrated dental implants.

#### 2.3.2. Intervention

The intervention was defined as regenerative surgical procedures for the treatment of PI incorporating BG materials in combination with a barrier membrane, following GBR principles.

#### 2.3.3. Comparison

The intervention was compared with regenerative surgical procedures involving the use of BG materials alone, without the adjunctive application of a barrier membrane.

#### 2.3.4. Outcomes

Primary outcomes were PPD reduction and radiographic MLB gain, while secondary outcomes included CAL gain, BOP, and membrane-related complications such as membrane exposure and wound dehiscence.

### 2.4. Search Strategy

An electronic literature search was conducted across four major databases: PubMed, Scopus, Google Scholar, and the Cochrane Library. The search strategy employed Medical Subject Headings (MeSH) terms and keywords related to PI, regenerative procedures, reconstructive treatments, and membrane applications. The search was limited to English-language publications published from January 1990 to 1 March 2024. Only studies published in English were included due to resource constraints and the lack of reliable translation tools for clinical terminology. While the use of translation services was considered, it was ultimately deemed impractical for ensuring accuracy in data extraction. The comprehensive search strategy used in PubMed was as follows: ((“peri-implantitis” OR “peri-implantitis” [MeSH terms] OR “peri-implant infection” OR “peri-implant disease*” OR “peri-implant defect” OR “peri-implant inflammation”) AND (“regenerative” OR “reconstructive” AND “surgery” OR “surgical” OR “membrane” OR “collagen” OR “PTFE”)), NOT (Review [publication type]). This search strategy was appropriately adapted for use in Scopus, the Cochrane Library, and Google Scholar using database-specific syntax.

To ensure comprehensive coverage of the literature, reference lists of all included articles were manually examined and a supplementary citation search was independently conducted by two authors to identify additional potentially eligible studies that might have been missed during the initial electronic database search.

Data extraction was performed independently by two review authors (Y.J.-C., Y.T.-J.) using a standardized data extraction form specifically designed to systematically collect information on study design characteristics, sample sizes, intervention details, outcome measures, and follow-up periods.

### 2.5. Study Selection, Assessment, and Agreement

Studies that did not meet the following inclusion criteria were excluded from the analysis: (1) Human prospective or retrospective follow-up studies and clinical trials, cohort studies, case–control studies, and case series with a minimum of 10 implants and at least 6 months of follow-up; (2) Comparative studies involving the use of membranes; (3) Clear definition of PI, including clinical and/or radiographic bone loss [1,2].

Exclusion criteria were as follows: (1) Studies in languages other than English; (2) Studies with inaccessible relevant data (e.g., inability to contact authors); (3) Studies on non-Ti surfaces; (4) Insufficient information regarding the topic; (5) No clear definition of PI.

The screening process was implemented in two sequential stages: initial evaluation of titles and abstracts, followed by comprehensive full-text review. Two authors (Y.J.-C., Y.T.-J.) independently assessed each study at both screening levels, and any disagreements were resolved through collaborative discussion. Final inclusion decisions were reached through consensus among all participating authors.

Among the 113 full-text articles retrieved, 98 were excluded for the following reasons: non-randomized study design (n = 44), animal study (n = 12), not relevant to the focused PICO question (n = 25), non-original research (n = 10), insufficient data reporting (n = 5), and lack of comparative groups (n = 2). These details are visually summarized in Figure 1.

In addition to clinical outcomes, the following data were extracted from each study: study design, number of implants and patients, type and material of BG and membrane, follow-up duration, intervention details (e.g., adjunctive therapies), and any reported adverse events or complications (Supplementary Table S1). In cases with missing or unclear data, such instances were reported as “not reported” or “unclear” in the extraction table.

### 2.6. Radiographic MLB Gain Assessment

Radiographic MLB gain was extracted as reported in the included studies. All 15 studies assessed peri-implant bone levels using standardized periapical radiographs. Most studies measured bone changes linearly in millimeters, typically from the implant shoulder or abutment junction to the most apical bone-to-implant contact. However, specific measurement method details (e.g., software-assisted vs. manual) were inconsistently reported across studies.

### 2.7. Risk of Bias Assessment

**Figure 2:**
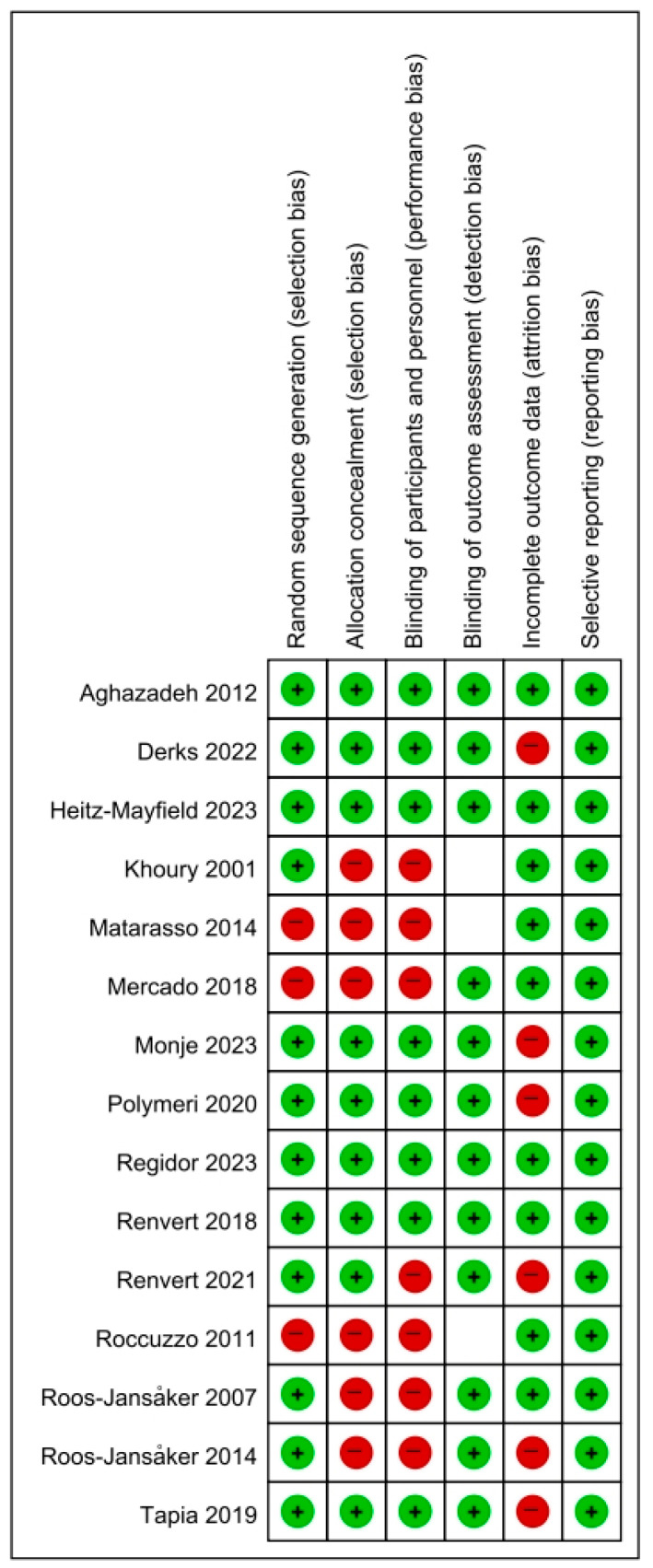
Summary of risk of bias (RoB) assessments for each included study across six domains, as evaluated by the review authors [9,10,12,13,14,15,16,17,18,19,20,21,22,23,24]. Green “+” indicates low RoB, red “–” indicates high RoB, and blank cells represent unclear risk.

The risk of bias (RoB) in the included studies was assessed with the Cochrane RoB tool (ver. 1.0) [11], evaluating domains such as randomization, blinding, and incomplete outcome data (Figure 2). Six domains were evaluated: random sequence generation, allocation concealment, performance bias, detection bias, attrition bias, and reporting bias.

The RoB was categorized into three levels: High: two or more criteria were not met or unclear; moderate: one criterion was not met; and low: all criteria were met. Two authors (Y.J.-C., Y.T.-J.) independently assessed all selected studies to ensure an unbiased evaluation.

**Figure 3:**
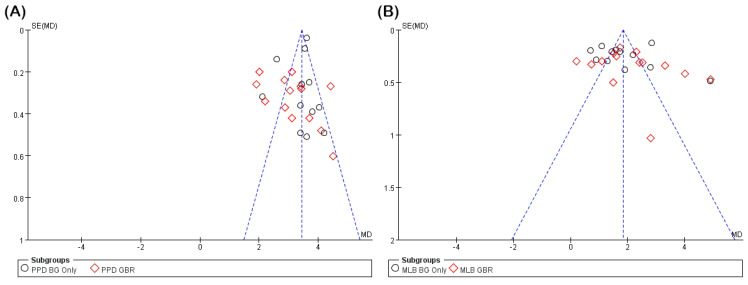
Funnel plots assessing publication bias using Egger’s test. (**A**) Funnel plot for PPD reduction, showing separate data points for BG only (○) and GBR (◇) subgroups. No significant asymmetry was observed in the BG group (*p* = 0.489), while a trend toward asymmetry was noted in the GBR group (*p* = 0.039). (**B**) Funnel plot for MLB gain. Egger’s test indicated statistically significant asymmetry in the BG group (*p* = 0.030), suggesting potential publication bias, while no asymmetry was detected in the GBR group (*p* = 0.217). In each funnel plot, the vertical dashed line indicates the summary effect estimate (mean difference), and the diagonal dashed lines represent the pseudo 95% confidence limits forming the expected funnel shape in the absence of publication bias. BG, bone graft; GBR, guided bone regeneration; MD, mean difference.

Risk of reporting bias, including publication bias, was assessed for the two main outcomes, PPD reduction and MLB gain, using both funnel plot visualization and Egger’s regression test (Figure 3). Funnel plot symmetry was visually inspected to evaluate small-study effects, and Egger’s test was performed separately for each subgroup (BG only and GBR) to detect potential asymmetry. Results were interpreted considering the number of studies and potential heterogeneity. Due to variability in reporting quality and outcome definitions, no formal assessment of evidence certainty (e.g., using the GRADE approach) was conducted. Although funding sources were reviewed where reported, inconsistent disclosure across studies precluded any formal analysis of funding-related bias.

### 2.8. Data Analysis

The meta-analysis was conducted using a random-effects model implemented in Review Manager (RevMan, version 5.4). For each outcome variable, including PPD reduction, radiographic MLB gain, and CAL gain, the mean difference (MD) and corresponding 95% confidence interval (CI) were calculated. Statistical heterogeneity across studies was assessed using the I^2^ statistic, with values above 50% interpreted as substantial heterogeneity. A *p*-value < 0.05 was considered statistically significant.

To assess the risk of publication bias, funnel plots were generated and visually inspected. In addition, Egger’s regression tests were conducted using R (metafor package) for both PPD and MLB outcomes, including subgroup analyses (BG only vs. GBR). Due to variability in study design and limited sample sizes, sensitivity analyses were not conducted. Evidence certainty was not formally assessed using the GRADE approach.

## 3. Results

### 3.1. Overview of Included Studies (Table 1)

A total of 331 records were produced by the first electronic database searches and manual reference screening. Following duplicate removal and systematic title/abstract screening, 111 full-text articles underwent detailed eligibility assessment. Of these, 15 studies fulfilled the predefined inclusion criteria and were incorporated into the final qualitative and quantitative analysis [9,10,12,13,14,15,16,17,18,19,20,21,22,23,24] (Figure 1).

The final study sample comprised eight randomized controlled trials (RCTs) [10,14,18,19,21,22,23,24] and seven prospective or retrospective cohort studies [9,12,13,15,16,17,20], with follow-up periods ranging from 6 to 60 months. The analysis encompassed clinical data from 560 patients who received surgical treatment for PI across all included studies.

All studies examined regenerative surgical interventions utilizing BG materials, with or without the adjunctive application of barrier membranes. Clinical outcomes were evaluated using standardized parameters including PPD reduction, CAL gain, and radiographic assessment of MLB gain.

Study design characteristics, sample sizes, types of BG materials and membranes employed, additional therapeutic interventions, and specific outcome measures are presented in detail in Supplementary Table S1.

### 3.2. Quality Assessment and Reporting Bias

The methodological quality of included studies was assessed using the Cochrane RoB tool (ver. 1.0), which evaluates six key domains: random sequence generation, allocation concealment, blinding of participants and personnel, blinding of outcome assessment, incomplete outcome data, and selective outcome reporting (Figure 2).

The assessment revealed substantial variation in study quality across evaluated domains. Random sequence generation demonstrated the most consistent performance, with 12 of 15 studies rated as low risk, indicating appropriate randomization protocols in the majority of investigations. Selective reporting was also adequately addressed, with 13 studies achieving low-risk ratings in this domain. Regarding incomplete outcome data, 11 studies were judged as low risk, reflecting generally acceptable management of participant attrition.

However, several methodological weaknesses were identified. Allocation concealment was frequently unclear or inadequate, with only six studies achieving low-risk ratings and the remainder demonstrating unclear or high-risk implementation. Blinding of participants and personnel, a critical factor for minimizing performance bias, was insufficiently described or omitted in most cases, resulting in 10 studies being rated as unclear or high risk. Blinding of outcome assessment showed relatively better implementation, with twelve studies rated as low risk. Nevertheless, three studies failed to provide sufficient methodological detail for evaluation of this domain and were consequently marked as “unclear”. Despite this limitation, overall bias judgments for these studies were determined based on the remaining assessable domains, consistent with established Cochrane guidance.

Based on the number of high-risk or unclear domains, the overall quality assessment classified six studies as high risk (≥2 domains at risk), five as moderate risk (only one domain at risk), and four as low risk across all evaluated domains. This considerable variability in methodological rigor represents an important consideration when interpreting the pooled outcomes of this systematic review.

To evaluate potential reporting bias, funnel plot analysis and Egger’s regression testing were conducted for both PPD and MLB outcomes (Figure 3). The PPD analysis revealed largely symmetrical funnel plots with no evidence of asymmetry in the BG group (*p* = 0.489) and only marginal significance in the GBR group (*p* = 0.039), suggesting minimal publication bias. Conversely, the MLB gain analysis indicated statistically significant asymmetry in the BG group (*p* = 0.030) and no asymmetry in the GBR group (*p* = 0.217). These findings suggest a potential risk of small-study effects or selective reporting, particularly within the MLB BG subgroup.

### 3.3. Clinical Outcomes of Studies

#### 3.3.1. PPD Reduction

**Figure 4:**
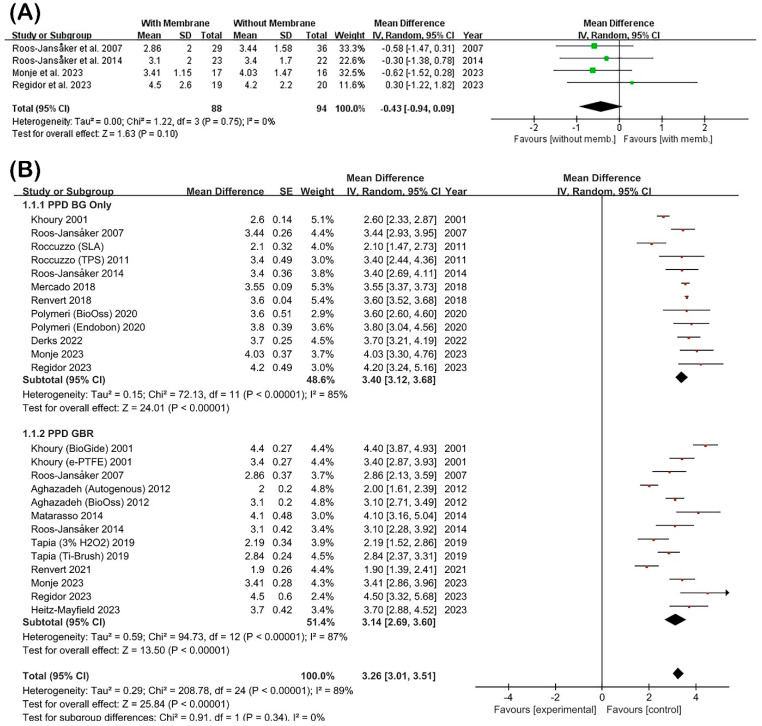
Forest plots for PPD reduction comparison between membrane and non-membrane groups [9,10,12,13,14,15,16,17,18,19,20,21,22,23,24]. (**A**) Meta-analysis of four studies directly comparing membrane and non-membrane groups, revealing no statistically significant difference in PPD reduction. (**B**) Pooled analysis of all included studies categorized by treatment type (BG only vs. GBR). Both subgroups demonstrated comparable clinical improvements, suggesting that membrane use does not offer additional benefit for PPD reduction.

PPD reduction was systematically evaluated across studies comparing regenerative treatment approaches that used barrier membranes with those that did not (Figure 4). The meta-analysis of directly comparative data (Figure 4A) included four studies that examined both treatment modalities within the same investigation. The pooled MD between membrane and non-membrane groups was −0.43 mm (95% CI: −0.94–0.09; *p* = 0.10), demonstrating no statistically significant difference between approaches. Notably, heterogeneity was minimal (I^2^ = 0%), indicating consistent treatment effects across the comparative studies.

The comprehensive pooled analysis incorporating all 25 treatment arms from the included studies (Figure 4B) demonstrated significant PPD reductions in both intervention categories. When BG material was utilized without membrane application, the mean PPD reduction achieved was 3.40 mm (95% CI: 3.12–3.68; I^2^ = 85%), with substantial heterogeneity observed between studies. In the membrane group, where BGs were combined with barrier membranes, the mean PPD reduction was 3.13 mm (95% CI: 2.71–3.55; I^2^ = 86%). The overall pooled estimate across both subgroups was 3.26 mm (95% CI: 3.01–3.51; I^2^ = 89%), confirming a statistically significant reduction in PPD across all included studies (*p* < 0.00001). However, subgroup analysis revealed no significant difference between treatment modalities (*p* = 0.34), indicating comparable effectiveness between membrane and non-membrane protocols.

These results demonstrate that regenerative surgical approaches consistently achieve clinically meaningful reductions in PPD regardless of membrane utilization. However, the adjunctive use of barrier membranes does not confer additional clinical benefit compared to BG-only treatment protocols.

#### 3.3.2. Radiographic MLB Gain

**Figure 5:**
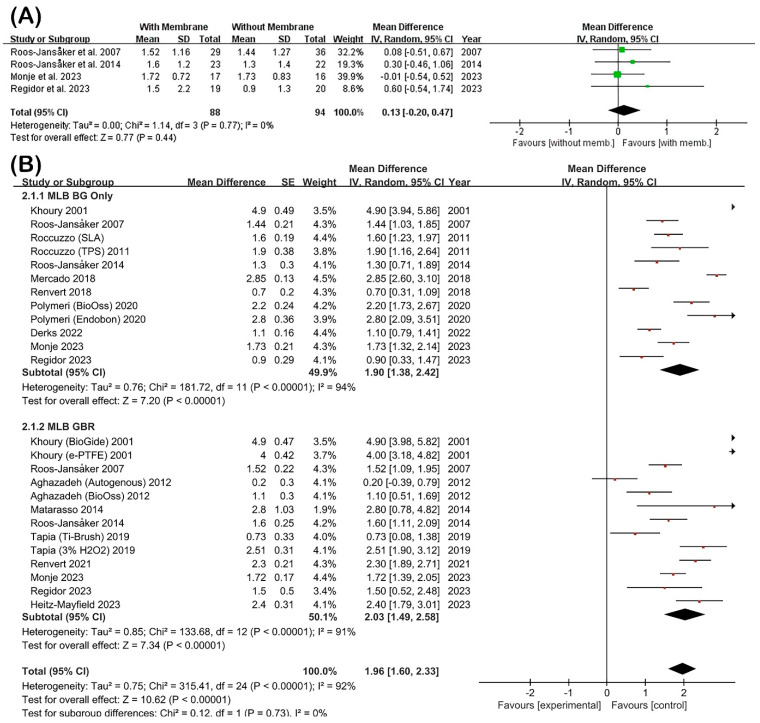
Forest plots for MLB gain in regenerative surgical treatment of PI [9,10,12,13,14,15,16,17,18,19,20,21,22,23,24]. (**A**) Meta-analysis of studies directly comparing membrane and non-membrane groups, showing no statistically significant difference in MLB gain. (**B**) Pooled analysis of all included studies categorized by treatment type (BG only vs. GBR). While both subgroups demonstrated significant MLB improvements, no meaningful difference was observed between them. High heterogeneity indicates variability in study protocols and outcomes.

Radiographic MLB gain was systematically assessed to determine the effectiveness of regenerative PI treatment modalities with and without barrier membrane application (Figure 5).

The subgroup meta-analysis examining four studies with direct comparisons between membrane and non-membrane approaches (Figure 5A) yielded a pooled MD of 0.13 mm (95% CI: −0.20–0.47; *p* = 0.44), demonstrating no statistically significant additional benefit from membrane utilization. The analysis exhibited negligible heterogeneity (I^2^ = 0%, tau^2^ = 0.00, chi^2^ = 1.14), indicating remarkable consistency across these comparative investigations.

The comprehensive pooled meta-analysis incorporating 25 treatment arms from 15 studies (Figure 5B) revealed statistically significant radiographic MLB in both treatment modalities. The non-membrane group achieved a weighted mean MLB gain of 1.90 mm (95% CI: 1.38–2.42; I^2^ = 94%; tau^2^ = 0.76), while the membrane group demonstrated a mean gain of 2.13 mm (95% CI: 1.59–2.67; I^2^ = 91%; tau^2^ = 0.85). The combined overall effect across all studies was 1.96 mm (95% CI: 1.60–2.33), with very high heterogeneity observed (I^2^ = 92%; chi^2^ = 315.41, *p* < 0.00001). Despite the greater mean gain in the membrane group, the test for subgroup differences failed to reach statistical significance (*p* = 0.55), indicating no clear therapeutic advantage of membrane application over BG alone.

These findings demonstrate that regenerative surgical treatment consistently produces clinically meaningful improvements in MLB. However, the adjunctive use of barrier membranes does not significantly enhance radiographic outcomes beyond those achieved through BG procedures alone.

#### 3.3.3. CAL Gain

**Figure 6:**
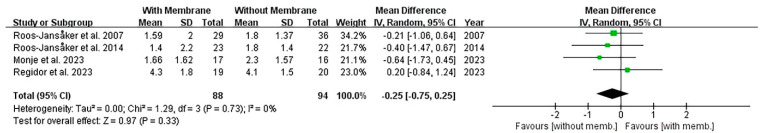
CAL gain forest plot. Meta-analysis of four studies comparing membrane and non-membrane groups showed no significant difference in CAL gain between treatment types. Results were consistent across studies, suggesting that membrane use does not enhance CAL outcomes [9,10,16,24]. Memb, membranes; CAL, clinical attachment level.

A meta-analysis of four studies directly comparing regenerative treatments with and without membrane use was conducted to assess CAL gain (Figure 6). The pooled analysis revealed an MD of −0.25 mm (95% CI: −0.75–0.25; *p* = 0.33), demonstrating no statistically significant advantage for membrane application compared to non-membrane protocols.

The analysis revealed excellent cross-study consistency, with negligible heterogeneity (I^2^ = 0%, tau^2^ = 0.00; chi^2^ = 1.29, df = 3; *p* = 0.73). Individual study results displayed small and variable differences in CAL gain, with none achieving statistical significance. The direction of effects was inconsistent—one study slightly favored membrane use, while others either favored non-membrane approaches or showed no meaningful difference.

These findings collectively indicate that the adjunctive use of membranes does not confer clinically meaningful improvements in CAL gain for regenerative PI treatment.

#### 3.3.4. Comparison of Membrane Type

**Figure 7:**
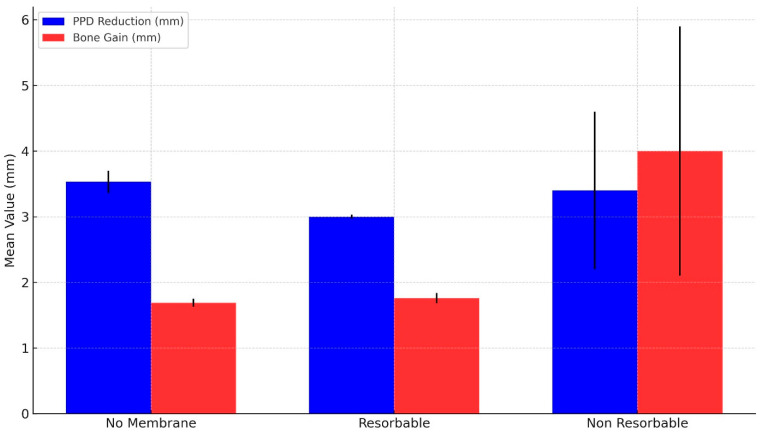
Comparison of weighted mean values of PPD reduction and MLB gain (mm) according to type of membrane used in GBR procedures. PPD reduction and MLB gain values are shown in blue and red, respectively. Error bars represent 95% CIs. For the non-resorbable membrane group, values were extracted from a single study; therefore, CIs reflect only within-study variance and should be interpreted with caution.

A subgroup analysis was performed to investigate the effect of membrane type—categorized as no membrane, resorbable membrane (e.g., collagen), and non-resorbable membrane (e.g., e-PTFE)—on clinical outcomes. Weighted mean values for PPD reduction and MLB gain were computed and compared among the three groups (see Table 2 and Figure 7).

The non-membrane group showed a mean PPD reduction of 3.53 mm (95% CI: 3.21–3.86) and MLB gain of 1.69 mm (95% CI: 1.56–1.81). Outcomes in the resorbable membrane group were comparable, with a mean PPD reduction of 3.00 mm (95% CI: 2.95–3.06) and MLB gain of 1.76 mm (95% CI: 1.61–1.92). This suggests that the addition of a resorbable membrane to a BG may not confer additional PPD benefit over bone grafting alone. Interestingly, the non-resorbable membrane group demonstrated the greatest mean PPD reduction of 3.40 mm (95% CI: 1.05–5.75), similar to the non-membrane group, but derived from a single study. The corresponding MLB bone gain in this group was 4.00 mm (95% CI: 0.28–7.72), also with a wide confidence interval indicating high variability and reduced interpretive certainty.

## 4. Discussion

### 4.1. Summary of Findings and Meta-Analytical Interpretation

The pooled results from our meta-analysis demonstrate that regenerative treatments, regardless of membrane use, led to substantial improvements in clinical outcomes such as PPD, radiographic MLB, and CAL. While both treatment modalities, BGs alone and BGs combined with membranes (GBR), achieved statistically significant improvements from baseline values, direct comparisons between approaches revealed no additional clinical benefit attributable to membrane application. This absence of superiority was consistently observed across all primary outcomes, highlighting the need to reassess the routine incorporation of membranes in regenerative PI therapy.

Our systematic review and meta-analysis found no statistically significant advantage of employing barrier membranes alongside BGs for the regenerative surgical management of PI. Clinical parameters including PPD, radiographic MLB, and CAL demonstrated significant improvement from baseline in both treatment groups, with no meaningful differences observed between membrane and non-membrane approaches. Supporting evidence from a 5-year comparative study showed PPD reduction of approximately 3.0–3.3 mm and MLB gain averaging 1.1–1.3 mm in both membrane and non-membrane groups, with an MD of only 0.4 mm (*p* = 0.24) [16]. Our meta-analytical findings consistently confirmed the absence of significant differences between groups across these critical parameters, suggesting that GBR using BGs alone can achieve clinical improvements comparable to those of combined BG and membrane techniques (Figure 4 and Figure 5).

### 4.2. Comparison with Previous Literature

These results correspond with recent RCT evidence. For instance, Monje et al. documented an approximate 77% disease resolution at 1 year after regenerative surgery utilizing bone allografts, with no enhancement in outcome rates upon the addition of collagen membranes [10]. The RCT revealed that patient-related factors, including low plaque scores and sufficient keratinized mucosa, were significantly superior predictors of successful healing compared to membrane utilization [10]. An umbrella review by Solderer and Schmidlin [25] similarly concluded that existing studies have not demonstrated enhanced clinical outcomes with the incorporation of a membrane, especially in defects enclosed by bony walls. Membranes were significantly linked to elevated complication rates, with reports indicating a 58% incidence of membrane exposure or dehiscence, thereby reducing any prospective advantage [26]. The convergent results from our meta-analysis, recent RCTs, and reviews indicate that the routine application of barrier membranes may not be universally justified in regenerative surgery for PI [10,25]. Collectively, these findings indicate that although regenerative surgery with BG is clinically effective for managing PI, the adjunctive use of membranes does not offer statistically significant improvements in outcomes such as PPD reduction, MLB gain, or CAL gain. These findings are consistent with the umbrella review, which also reported limited evidence supporting the clinical benefit of membranes and highlighted high complication rates, such as exposure and dehiscence [25]. To further investigate the potential role of membrane characteristics in influencing outcomes, we conducted a subgroup analysis comparing three groups: no membrane, resorbable membranes, and non-resorbable membranes. The pooled results showed that both the no membrane and resorbable membrane groups yielded similar outcomes, with mean PPD reductions of 3.53 mm and 3.00 mm and mean MLB gains of 1.69 mm and 1.76 mm, respectively. In contrast, the non-resorbable membrane group exhibited higher MLB gain (4.0 mm), although this result was derived from a single study and should be interpreted with caution due to its wide CI. These findings suggest that resorbable membranes do not offer significant additional benefit over BG-only approaches in typical cases and may guide clinicians to reserve membrane use for specific indications. They also highlight the importance of selecting membranes based on material properties and the clinical context. Therefore, routine membrane use should not be assumed as a standard of care but, rather, considered selectively based on defect characteristics (e.g., wall number or containment) and patient-specific risk profiles (e.g., plaque control, soft tissue thickness). This aligns with prior reviews emphasizing a personalized, case-based approach over uniform protocol adoption [10,25,26].

Due to the absence of evident advantages associated with barrier membranes, clinicians must meticulously evaluate defect morphology and overarching treatment objectives when determining their necessity in PI surgery. In contained intrabony defects characterized by three- or four-wall osseous support, a BG alone is frequently adequate to stabilize the defect and facilitate MLB gain [27]. In such instances, incorporating a membrane may provide minimal benefit while increasing the likelihood of wound dehiscence and infection due to membrane exposure [25,26,28]. Conversely, membranes may be utilized in non-contained or extensive (one- or two-wall) defects where BG material would otherwise be unstable or susceptible to soft tissue invagination. Consensus recommendations advocate for the utilization of membranes predominantly in intricate, non-autonomous defects to facilitate space maintenance [25]. It is important to acknowledge the paucity of direct clinical evidence showing improved outcomes in non-contained defects with membranes. Consequently, the choice to employ a membrane must be personalized, considering defect morphology, soft tissue density, and patient risk factors (e.g., smoking, oral hygiene). Effective infection control, comprehensive debridement, and implant surface decontamination are essential prerequisites for healing with all regenerative methods.

Nevertheless, in contrast to the present review’s main findings, some studies have reported that using barrier membranes may yield favorable clinical outcomes under certain conditions. For instance, Heitz-Mayfield et al. demonstrated significant improvements in CAL and radiographic MLB gain when resorbable collagen membranes were combined with xenogeneic BGs [23]. Similarly, Regidor et al. reported greater bone regeneration percentages in non-contained peri-implant defects treated with barrier membranes [24]. Although these studies appear to support the beneficial effects of membranes, their findings are largely context-dependent, often relying on specific surgical conditions or adjunctive therapies (e.g., the use of biologics, antibiotics, or wide bands of keratinized mucosa), making the attribution of clinical improvements solely to membrane application difficult. This interpretation aligns with the findings from Table 1 in our study, where comparable levels of PPD reduction and MLB gain were observed regardless of membrane use. These results suggest that clinical outcomes may be more influenced by membrane type, defect morphology, and additional procedural factors, such as flap design, membrane stabilization techniques, suturing methods, and implant surface decontamination protocols, than by membrane utilization. In particular, the distinction between resorbable and non-resorbable membranes appears clinically relevant. That is, non-resorbable membranes (e.g., expanded polytetrafluoroethylene (e-PTFE)) have shown promising regenerative outcomes in early studies but have also been associated with higher rates of membrane exposure and infection, leading to inconsistent clinical results, as shown in Table 1 [12,16]. Conversely, resorbable membranes (e.g., collagen-based) offer better handling and lower complication rates, but their limited space-maintaining capacity may reduce effectiveness in wide or non-contained defects. As such, the membrane’s mechanical properties and the defect’s anatomical characteristics must be considered simultaneously when evaluating potential benefits.

### 4.3. Clinical Implications and Decision Making

To overcome these clinical limitations, alternative adjunctive interventions have demonstrated potential in PI management, especially in enhancing infection control [29,30,31]. One promising approach involves the local application of antimicrobial agents. Among the studies included in this meta-analysis, only one reported the use of local doxycycline gel in combination with enamel matrix derivative (EMD) as an adjunctive antimicrobial therapy [17]. The remaining studies did not employ local antibiotics, thereby limiting subgroup analyses based on antimicrobial intervention. A recent systematic review and meta-analysis by Toledano et al. [29] revealed that the localized application of antibiotics (e.g., minocycline or doxycycline gel) as an adjunct to PI treatment resulted in a significantly greater reduction in PPD and an increased likelihood of BOP reduction compared to control groups. The enhancements were realized without negative consequences, underscoring that antimicrobial adjuncts can improve the inflammatory metrics of PI therapy. Conversely, the application of membranes focuses on promoting regeneration rather than diminishing bacterial load, and the evidence indicates that membranes do not confer additional PPD reduction advantages compared to conventional regenerative surgery [25,27]. This distinction emphasizes a crucial clinical aspect. The integration of thorough debridement with antimicrobial approaches (local or systemic antibiotics, antiseptics) may be more vital for the initial resolution of PI than the mere presence of a membrane [32,33]. The successful eradication of bacteria establishes the foundation for any regenerative therapy to be effective. 

An additional significant adjunctive strategy is the modification of implant surfaces during surgical procedures. Methods like implantoplasty (mechanical refinement of implant threads) have been utilized to diminish the surface roughness that facilitates biofilm accumulation [15,34,35,36]. A recent study demonstrated that implantoplasty, frequently paired with apically positioned flaps or osteoplasty, can arrest disease progression and enhance clinical outcomes by promoting plaque management [37]. They attained a significant treatment success rate without membranes by removing peri-implant pockets and refining the implant surface, contingent upon effective postoperative plaque control. This review indicated that all treatments, whether regenerative or resective surgery, resulted in substantial enhancements in PPD and BOP from baseline, underscoring the importance of mechanical disruption of the biofilm and thorough decontamination for therapeutic success. Monje et al. [10] further corroborated this, revealing that implants with inadequate plaque control continued to experience bone loss despite regenerative surgery, while implants in patients with excellent oral hygiene remained stable. Consequently, clinical decision making must prioritize strategies that guarantee a sterile, biologically compatible implant surface (utilizing mechanical methods, chemical agents such as citric acid or chlorhexidine, and/or laser therapy) and promote effective patient plaque management. In addition to these surface-focused strategies, the role of barrier membranes also warrants critical consideration. Based on current evidence, the routine use of barrier membranes in regenerative PI surgery cannot be universally justified. However, in specific clinical scenarios, particularly non-contained defects with limited (e.g., one to two) bony walls, the adjunctive use of resorbable membranes may provide benefits for space maintenance and BG stabilization. Membrane application should be implemented selectively, with careful consideration of defect configuration, soft tissue biotype, and patient-related factors, including oral hygiene compliance and smoking status. Resorbable collagen membranes may be preferred over non-resorbable alternatives due to their lower exposure risk and superior handling characteristics. Nevertheless, the decision to incorporate a membrane should be individualized rather than protocol-driven, pending the availability of further defect-specific RCTs.

Adjunctive interventions, including EMD or growth factors, have been explored in regenerative PI treatment [17]. Although certain case series have indicated positive bone augmentation with combinations such as EMD + BG + antibiotic, the additional benefit of biologics compared to BG-only protocols remains uncertain [32]. Likewise, laser-assisted debridement (e.g., Er:YAG or photodynamic therapy) has been investigated as a complement to traditional surgery [38,39]. Lasers can significantly diminish microbial loads and prompt initial enhancements; however, controlled trials indicate no long-term benefits in PPD or CAL from laser decontamination compared to conventional open-flap debridement [40].

### 4.4. Limitations of the Evidence and Methodological Considerations

Despite the comprehensive analysis, several limitations in the existing evidence must be acknowledged. First, significant heterogeneity exists among studies regarding defect morphology, surgical technique, and materials used. That is, the trials varied in the containment of defects and in the types of BGs (xenograft, allograft, etc.), membrane materials (native collagen, non-resorbable, absence of membrane), and techniques for implant surface decontamination (mechanical curettage, implantoplasty, chemical agents, lasers, etc.) utilized, making direct comparison difficult. Systematic reviews have identified a lack of consensus regarding the optimal regenerative strategy, as various combinations of BGs and membranes have been employed with inconsistent outcomes [41]. The resulting clinical heterogeneity frequently produces significant statistical heterogeneity in meta-analyses, requiring careful interpretation of aggregated results.

Another limitation relates to the variability in implant surface decontamination protocols employed across the included studies. While some investigations utilized mechanical debridement or curettage, others incorporated chemical agents or employed laser-assisted decontamination techniques. This heterogeneity in biofilm removal strategies may significantly influence regenerative outcomes by affecting the potential for successful reosseointegration. Given that effective decontamination is necessary for successful bone regeneration, variations in these procedures may confound the comparative evaluation of membrane efficacy. Future studies should prioritize the standardization of decontamination protocols or stratify outcomes according to the specific technique employed to minimize bias and enhance comparability.

Secondly, numerous studies exhibited limited sample sizes and follow-up durations. Most RCTs in this domain encompassed approximately 20–50 implants and monitored patients for 12 months, which may be insufficient to identify differences in hard tissue outcomes or to detect late complications. Longitudinal data spanning 3 to 5 years or more are limited, although we incorporated several studies with 36- or 60-month follow-ups into our review. Furthermore, only 4 out of the 15 included studies had follow-up durations of 36 months or longer, and none exceeded 5 years. As such, the long-term durability of regenerated bone and sustained clinical success following membrane application remain inadequately evaluated [42]. Without sufficient long-term data, definitive conclusions about membrane stability, risk of relapse, or true reosseointegration cannot be drawn. Future studies with standardized, extended follow-up periods are essential to clarify these critical outcomes [43]. It is worth noting that some studies did not randomize patients to treatments or used uneven sample sizes, which introduces potential bias, highlighting the necessity for more rigorous, adequately powered RCTs.

Third, inconsistency in the definitions of outcomes and criteria for success among studies was observed. Certain authors delineated treatment success exclusively through PPD reduction and radiographic MLB gain, whereas others incorporated the absence of BOP/suppuration and implant survival into their success criteria. PPD measurements concerning implants are sensitive to technique and are reported less frequently than those in conventional periodontitis research. Furthermore, radiographic MLB gain does not signify actual reosseointegration; in the absence of histological evidence, it remains uncertain whether the regenerated bone is completely integrated with the implant surface [44]. The utilization of various indices and thresholds for clinically significant improvement complicates the aggregation of data and the establishment of a universal benchmark for success.

Ultimately, publication and methodological biases must be considered. Although our meta-analysis and others have underscored the absence of a substantial membrane effect, some authors might have chosen not to publish negative results. In contrast, numerous studies incorporated adjunctive therapies (e.g., BGs, diverse decontamination techniques, and antibiotics) in both the experimental and control groups, which may obscure the distinct impact of a membrane. Variations in surgical proficiency and experience, along with postoperative care protocols, further complicate the situation. Overall, existing evidence suggests that membranes do not significantly alter the course of PI regeneration; however, the previously mentioned limitations diminish the robustness of any recommendations. In an initial review, Renvert et al. [45] highlighted that predictable PI treatment is difficult and frequently necessitates supplementary interventions beyond mechanical cleaning, a conclusion that continues to hold given the heterogeneity and uncertainties in data.

### 4.5. Evolution of Membrane Use and Future Directions

Over the past three decades, the application and understanding of membranes in regenerative PI surgery have changed dramatically. Early investigations during the 1990s and early 2000s predominantly utilized non-resorbable membranes, particularly e-PTFE, and emphasized space maintenance and mechanical defect stabilization [46,47]. However, these early approaches were frequently complicated by membrane exposure and secondary infections, particularly in non-contained or supracrestal defect configurations. Contemporary research has increasingly favored resorbable collagen-based membranes, which demonstrate superior biocompatibility and clinical handling characteristics [23,48,49]. The field has further advanced with the development of novel membrane technologies incorporating bioactive agents such as antibiotics and growth factors [29,32,33,50]. These innovations aim to extend therapeutic benefits beyond traditional physical barrier function to actively promote regenerative processes. Despite these technological advances, robust clinical evidence supporting the superiority of newer membrane systems remains limited [7,18].

A critical gap exists in the availability of long-term RCTs that systematically compare different membrane types under standardized clinical protocols. This limitation is particularly pronounced regarding the relationship between membrane selection and defect [51]. Future research priorities should encompass several key areas. Stratified treatment approaches that tailor membrane selection to specific defect characteristics require systematic investigation. Biologically active and antimicrobial membranes warrant evaluation in larger-scale human trials with adequate statistical power [23,52]. Additionally, comprehensive long-term follow-up studies are essential to assess the sustainability of regenerative outcomes and the durability of reosseointegration processes. These research directions will be fundamental in establishing evidence-based guidelines for membrane selection and application in regenerative PI therapy.

Future research must address the aforementioned limitations and investigate strategies to enhance regenerative outcomes in the management of PI. Addressing these limitations requires well-designed studies with uniform treatment protocols and outcome measures. This standardization would enable more significant comparisons across studies and meta-analyses, diminishing heterogeneity and bias. Considering the crucial influence of defect configuration on regenerative outcomes [25], clinical trials should stratify results by defect type or only include particular defect morphologies. Distinct studies examining contained versus non-contained defects could elucidate whether membranes confer a genuine advantage in one context compared to another. A controlled trial focusing solely on non-contained, extensive defects could help determine whether membrane application improves defect fill or enhances clinical stability. Investigations into advanced biomaterials, such as membranes infused with growth factors or antimicrobials, or 3D-printed scaffolds tailored to intricate defect geometries, may significantly enhance regenerative outcomes. Preliminary investigations utilizing autologous platelet concentrates (platelet-rich fibrin) have been conducted [50]; however, one RCT indicated that platelet-rich fibrin membranes produced worse outcomes than collagen membranes. Nevertheless, the integration of biological enhancers with barrier functions continues to be a promising approach. Stringent preclinical and clinical trials are essential to evaluate whether these innovations can produce superior enhancement in bone regeneration around implants compared to existing BGs and membranes.

In summary, although barrier membranes are frequently utilized in regenerative PI surgery, existing evidence does not substantiate a notable clinical advantage for their regular application compared to BG alone. Based on the current meta-analysis, regenerative procedures without membranes demonstrated a mean PPD reduction of approximately 3.3 mm and an average MLB gain of nearly 2.0 mm, results comparable to those achieved with membrane use. However, no statistically significant benefit of membranes was observed for any of the measured outcomes, including CAL. Despite the current lack of strong evidence supporting universal membrane use, select studies suggest potential benefits in specific indications. These findings warrant further validation in well-controlled, defect-specific trials to clarify the clinical scenarios in which membranes may offer meaningful regenerative advantages. Attaining and sustaining implant disinfection and an optimal oral environment seem to be the critical determinants for successful PI outcomes. Continuous high-quality research encompassing rigorously controlled trials and longitudinal data will be crucial for enhancing surgical protocols. Collectively, these efforts will guide clinicians toward more predictable, evidence-based strategies for managing PI and preserving peri-implant tissue health.

## 5. Conclusions

PI management is undergoing a paradigm shift, as accumulating evidence challenges the routine application of barrier membranes in regenerative surgical therapy. In this systematic review and meta-analysis, the adjunctive use of membranes did not provide statistically significant benefits in key clinical outcomes such as PPD reduction or CAL gain, while MLB gains were comparable to those achieved with bone grafts alone. These findings suggest that membranes should be selective rather than routine, but considered selectively based on defect configuration, tissue biotype, and individual patient risk factors. This individualized approach may help optimize clinical outcomes while reducing surgical complications and costs. Moving forward, clinical strategies should emphasize evidence-based decision making and minimal intervention. Innovations such as biologically active membranes, enhanced implant surface designs, and standardized regenerative protocols hold promise in improving the predictability and efficiency of PI treatment. Ultimately, sustained clinical success will depend on integrating scientific advancements with patient-centered care principles.

## Figures and Tables

**Table 1:** Probing pocket depth (PPD) reduction and marginal bone level (MLB) gain in regenerative treatment of peri-implantitis (PI) after 12 months.

Author	Year	Graft Material	Membrane	Other Factors	Defect Type	Radiograph Type and Evaluation Method	Mean ± SD		Sample Size	SE	
							PPD Reduction (mm)	MLB Gain (mm)		PPD Reduction	MLB Gain
Khoury [12]	2001	Autogenous	No		Intrabony	Periapical	2.6 ± 0.5	4.9 ± 1.7	12	0.14	0.49
			e-PTFE (Gore-Tex)			Linear Measurement (Manual)	3.4 ± 1.2	4 ± 1.9	20	0.27	0.42
			Bio-Gide				4.4 ± 0.8	4.9 ± 1.4	9	0.27	0.47
Roos-Jansåker [9]	2007	Algipore	No		N/A	Periapical	3.44 ± 1.58	1.44 ± 1.27	36	0.26	0.21
			Osseoquest			N/A	2.86 ± 2	1.52 ± 1.16	29	0.37	0.22
Roccuzzo [13]	2011	Bio-Oss collagen	No	TPS	Crater-Like	Periapical	2.1 ± 1.2	1.6 ± 0.7	14	0.32	0.19
				SLA		N/A	3.4 ± 1.7	1.9 ± 1.3	12	0.49	0.38
Aghazadeh [14]	2012	Autogenous	OsseoGuard		≥ 3 mm Angular Peri-Implant	Periapical	2 ± 0.2	0.2 ± 1.4	22	0.04	0.3
		Bio-Oss				Software-Assisted	3.1 ± 0.2	1.1 ± 1.43	23	0.04	0.3
Matarasso [15]	2014	Bio-Oss	Bio-Gide		Intrabony and Suprabony	Periapical	4.1 ± 1.6	2.8 ± 3.4	11	0.48	1.03
						Software-Assisted					
Roos-Jansåker [16]	2014	Algipore	No		1-Wall (14.6%) 2-Wall (29.2%) 3-Wall (43.8%) Circumferential (10.4%) Not Classified (2.1%)	Periapical	3.4 ± 1.7	1.3 ± 1.4	22	0.36	0.30
			Osseoquest			N/A	3.1 ± 2	1.6 ± 1.2	23	0.42	0.25
Mercado [17]	2018	Bio-Oss collagen	No	EMD, Doxy	Crater-Like or Circumferential	Periapical	3.55 ± 0.5	2.85 ± 0.73	30	0.09	0.13
						Linear Measurement (Manual)					
Renvert [18]	2018	Endobon	No		Crater-Like	Periapical	3.6 ± 0.2	0.7 ± 0.9	21	0.04	0.34
						Software-Assisted					
Tapia [19]	2019	BoneCeramic	Cytoplast (Resorbable)	3% H_2_O_2_	Intrabony	Periapical	2.19 ± 1.31	0.73 ± 1.26	15	0.34	0.33
				Ti-Brush		Software-Assisted	2.84 ± 0.93	2.51 ± 1.21	15	0.24	0.31
Polymeri [20]	2020	Bio-Oss	No		Intrabony	Periapical	3.6 ± 1.7	2.2 ± 0.8	11	0.51	0.24
		Endobon				Software-Assisted	3.8 ± 1.4	2.8 ± 1.3	13	0.39	0.36
Renvert [21]	2021	Bio-Oss	Bio-Gide		Intrabony	Periapical	1.9 ± 1.5	2.3 ± 1.2	34	0.26	0.21
						Software-Assisted					
Derks [22]	2022	Bio-Oss collagen	No		≥ 3 mm Circumferential and No Minimum Number of Walls	Periapical	3.7 ± 2.1	1.1 ± 1.4	72	0.25	0.16
						Software-Assisted					
Monje [10]	2023	Bio-Oss	No		Intrabony and Suprabony	Periapical	4.03 ± 1.47	1.73 ± 0.83	16	0.37	0.21
		Allograft	RTM			Linear Measurement (Manual)	3.41 ± 1.15	1.72 ± 0.72	17	0.28	0.17
Heitz-Mayfield [23]	2023	Bio-Oss	Bio-Gide		Intrabony	Periapical and CBCT	3.7 ± 1.9	2.4 ± 1.4	20	0.42	0.31
						Software-Assisted					
Regidor [24]	2023	Bio-Oss collagen	No		Intrabony (Minimum 2 Walls)	Periapical	4.2 ± 2.2	0.9 ± 1.3	20	0.49	0.29
			Bio-Gide			Software-Assisted	4.5 ± 2.6	1.5 ± 2.2	19	0.60	0.50

SD, standard deviation; SE, standard error; EMD, enamel matrix derivative; Doxy, doxycycline; TPS, titanium plasma-sprayed surface; SLA, sandblasted, large-grit, acid-etched surface; RTM, resorbable tissue matrix; e-PTFE, expanded polytetrafluoroethylene.

**Table 2:** Comparison of weighted mean PPD reduction and MLB gain (mm) according to membrane type used in regenerative procedures. Values are based on pooled data from multiple studies, except for the non-resorbable membrane group, which is derived from a single study.

	PPD Reduction (mm)		MLB Gain (mm)		Number of Studies	Total Sample Size
	Weighted Mean	95% CI	Weighted Mean	95% CI		
No Membrane	3.53 ± 0.17	[3.21, 3.86]	1.69 ± 0.06	[1.56, 1.81]	10	279
Resorbable	3 ± 0.03	[2.95, 3.06]	1.76 ± 0.08	[1.61, 1.92]	10	237
Non-resorbable	3.4 ± 1.2	[1.05, 5.75] *	4 ± 1.9	[0.28, 7.72] *	1	20

Values are presented as mean ± SE with 95% CI shown in brackets. * CI calculated from a single study; not from pooled data.

## Data Availability

No new data were created or analyzed in this study. Data sharing is not applicable to this article.

## Supplementary Materials

The following supporting information can be downloaded at: https://www.mdpi.com/article/10.3390/jfb16070262/s1, Table S1: Summary of included studies reporting on PI regenerative procedures, including author, year, graft materials, membrane types, radiographic evaluation methods, defect types, clinical outcomes (PPD reduction and MLB gain), and sample size.

## Abbreviations

BG, bone graft; PPD, probing pocket depth; CAL, clinical attachment level; BOP, bleeding on probing; MLB, marginal bone level; GBR, guided bone regeneration; MD, mean difference; CI, confidence interval; RCT, randomized controlled trial; RoB, risk of bias; RevMan, Review Manager; PRISMA, Preferred Reporting Items for Systematic Reviews and Meta-Analyses; PROSPERO, Prospective Register of Systematic Reviews; MeSH, Medical Subject Headings; MB, membrane; PI, Peri-implantitis; EMD, Enamel Matrix Derivative; TPS, titanium plasma-sprayed surface; SLA, sandblasted, large-grit, acid-etched surface; e-PTFE, expanded polytetrafluoroethylene.
